# A Collection of Useful Nuisance Compounds (CONS) for Interrogation of Bioassay Integrity

**DOI:** 10.1021/jacsau.4c00851

**Published:** 2024-11-25

**Authors:** Huabin Hu, Xiangyan Yi, Lian Xue, Jonathan B. Baell

**Affiliations:** † Science for Life Laboratory, Department of Cell and Molecular Biology, Uppsala University, BMC, Box 596, Uppsala SE-751 24, Sweden; ‡ Medicinal Chemistry, Monash Institute of Pharmaceutical Sciences, Monash University, Parkville, Victoria 3052, Australia

**Keywords:** PAINS, nuisance compounds, assay troubleshooting, assay integrity, assay interference, high-through screening, false positives

## Abstract

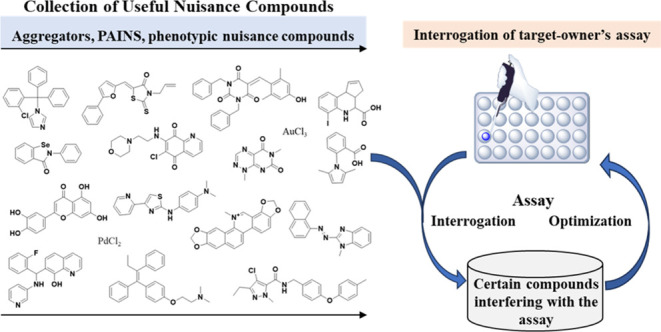

High-throughput screening (HTS) is a crucial technique for identifying potential hits to fuel drug discovery pipelines. However, this process naturally concentrates nuisance compounds that are not optimizable yet signal positively in a convincing manner. To be able to understand what types of nuisance compounds a particular assay is sensitive to, would be of great utility in being able to prioritize progressable over nonprogressable screening hits. In this study, we present a carefully compiled set of over 100 nuisance compounds that are known to interfere with assay readouts in either phenotypic or target-based screenings. Readily accessible in an assay-ready screening plate, we believe this nuisance compound set will be of great interest to the research community, helping to establish high-quality HTS assays and identify promising, optimizable hits.

## Introduction

Screening collections of compounds in bioassays is a highly successful approach to identify those with an intended bioactivity–so-called screening hits. However, sets of screening hits are typically dominated by compounds that may signal in the bioassay of interest, but do so in a manner that represents a compound that cannot be usefully optimized neither should be progressed.

Nuisance compounds are agnostic of the screening method, whether target-based (experimental or virtual) or phenotypic. These compounds may interfere with assay photometry, they may form aggregates, or they may be reactive.^1^ Reactivity can manifest in a variety of ways, from redox cycling to metal chelation, to electrophilicity toward biological nucleophiles. Sometimes a chemotype can be regarded as a nuisance class simply because it tends to be contaminated with impurities that promiscuously signal in bioassays. Compound classes that display a multitude of subversive mechanisms are called PAINS (pan-assay interference compounds). Such behavior is relevant to target-based assays as well as phenotypic assays. For the latter there are additional nuisance behaviors, such as membrane perturbation or binding to intrinsic cell machinery proteins such as tubulin.

The importance of being able to recognize these nuisance compounds in order to focus on those that can be optimized, is often not appreciated. Although electronic filters can flag PAINS compounds, there are several reasons why this in itself may be insufficiently comprehensive.^2^ Use of detergent can help to minimize interference by colloidal aggregates, and well-designed counter screens can be used to exclude assay technology interference compounds. However, PAINS that may be on-target through reactivity mechanisms will escape such interrogation. Access to a defined set of the most problematic nuisance compounds would be of great service to the drug discovery community, which researchers could process through their assays of interest in order to understand how susceptible those assays are to identifying subversive compounds, and what sort of compound types these are.

Here we present a defined set of 103 compounds, comprised of PAINS and other nuisance compounds, that we have made readily accessible in assay-ready screening format. A brief description of each nuisance compound class represented is provided below.

## Results and Discussion

### Colloidal Aggregates

**Figure 1:**
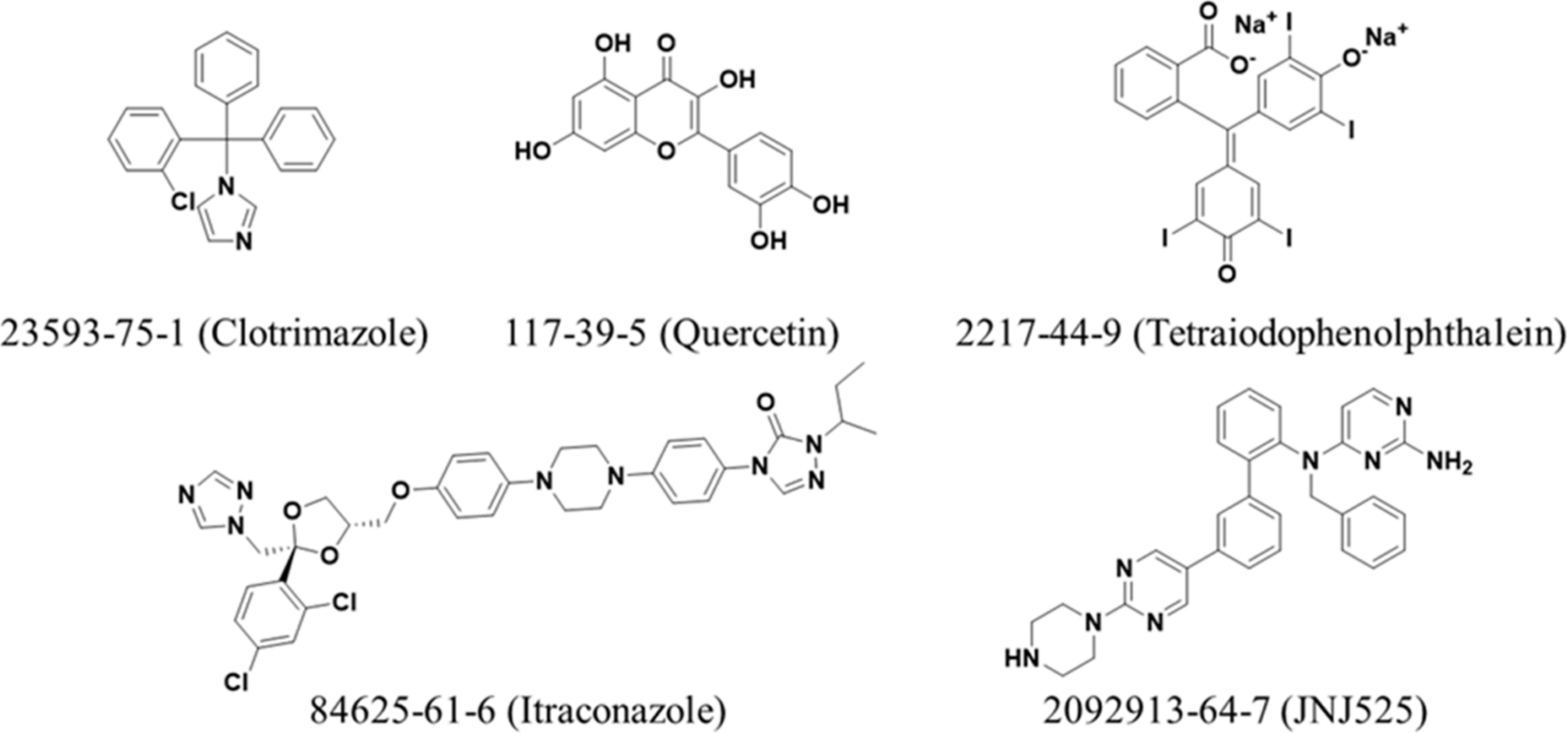
Representative colloidal aggregate-forming compounds. Displayed are common problematic compounds that disrupt assay readouts through colloidal aggregation mechanism, for which structural divergence is remarkable. The CAS number for each compound is provided along with its name in parentheses.

Certain compounds form colloidal aggregates, that interfere with protein signaling. For these compounds, inhibition based on aggregation may frequently occur at micromolar concentrations, resulting in the formation of colloidal aggregates ranging from 50 to over 500 nm in radius.^3^ Fortunately, inclusion of detergent can facilitate destruction of these aggregates and the associated bioactivity, but for researchers who may be unaware of this remedy, we include five classic colloidal aggregate-forming compounds (Figure 1). For instance, clotrimazole, an antifungal agent, has been found at micromolar concentrations to inhibit three unrelated model enzymes, namely β-lactamase, chymotrypsin, and malate dehydrogenase, through an aggregation-based mechanism.^4^ It was subsequently found that these colloidal aggregates could also modulate GPCR signaling in cell-based assays.^3^ Quercetin, one among a larger cohort of nuisance secondary metabolite phenols,^5^ can also function as a promiscuous, aggregation-based inhibitor by forming colloidal aggregates.^4,6^ Tetraiodophenolphthalein (TIPT) has been studied for its ability to coaggregate with Congo Red, conferring higher stability compared with single compound colloidal aggregates and possessing the capability of capturing and releasing active proteins.^7^ The traditional mechanism of aggregation-induced inhibition suggests that targeted proteins may adhere to the surface of the aggregate, leading to protein sequestration or denaturation.^8^ However, some compounds may act via inhibiting protein–protein interactions rather than inducing protein inactivation through aggregation. For instance, JNJ525, a small-molecule inhibitor of TNFα for cancer treatment, forms aggregates that can displace one of the TNFα subunits, subsequently triggering TNFα quaternary structure rearrangement, ultimately interfering the formation of TNFα complexes with TNFR1 and TNFR2.^9^ Molecules behaving as colloidal aggregation-based bioassay signal modulators may be widespread in drug discovery screening. Of immediate note is the structural diversity represented in such compounds (Figure 1), giving rising to tools such as Aggregate Advisor (http://advisor.bkslab.org/). This *in silico* resource identifies compounds that are similar to known aggregators and that therefore may have a propensity to form colloidal aggregates.

### PAINS

Certain classes of compounds can be regarded as PAINS. We have discussed the behavior of these compounds at length and for further details behind all chemotypes, the reader is referred to the underlying publications.^5,10,11^ Here we have focused just on the most common and well-recognized PAINS that are validated as promiscuous across a variety of independent and different methods (Table 1).^2^

**Table 1:**
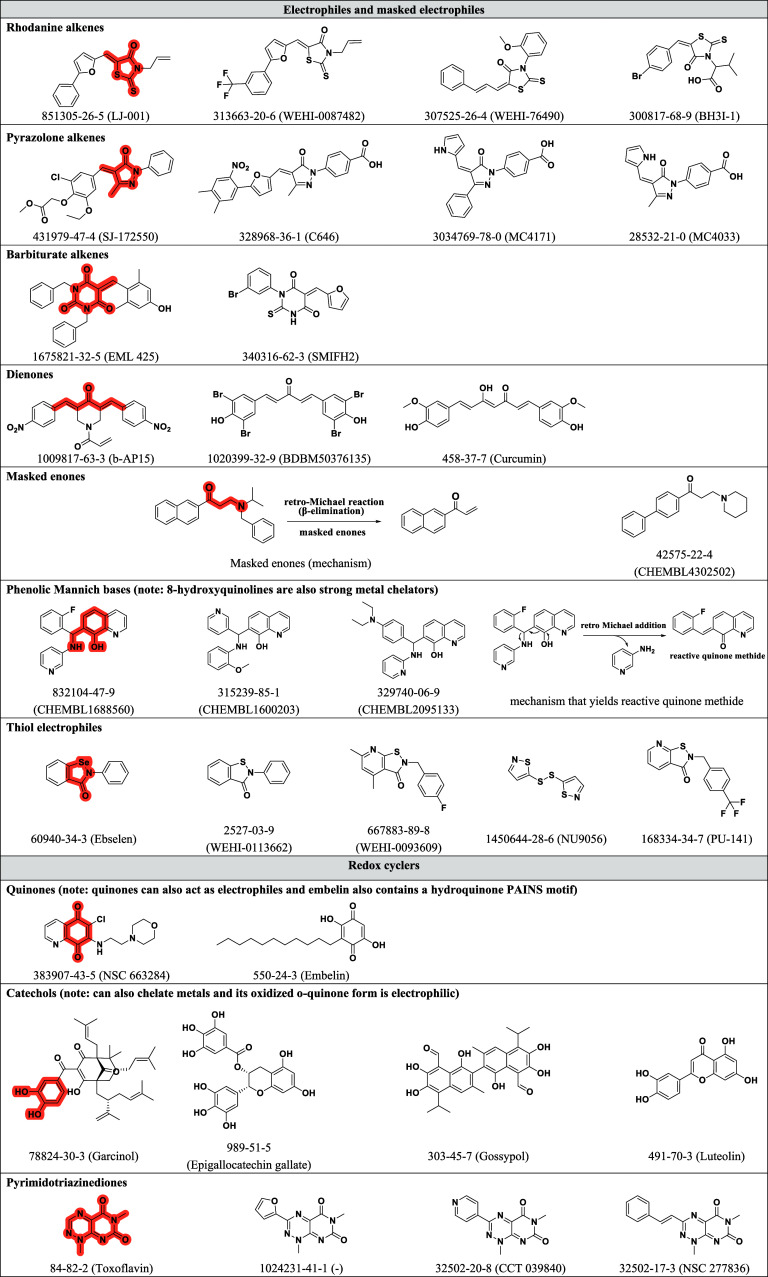

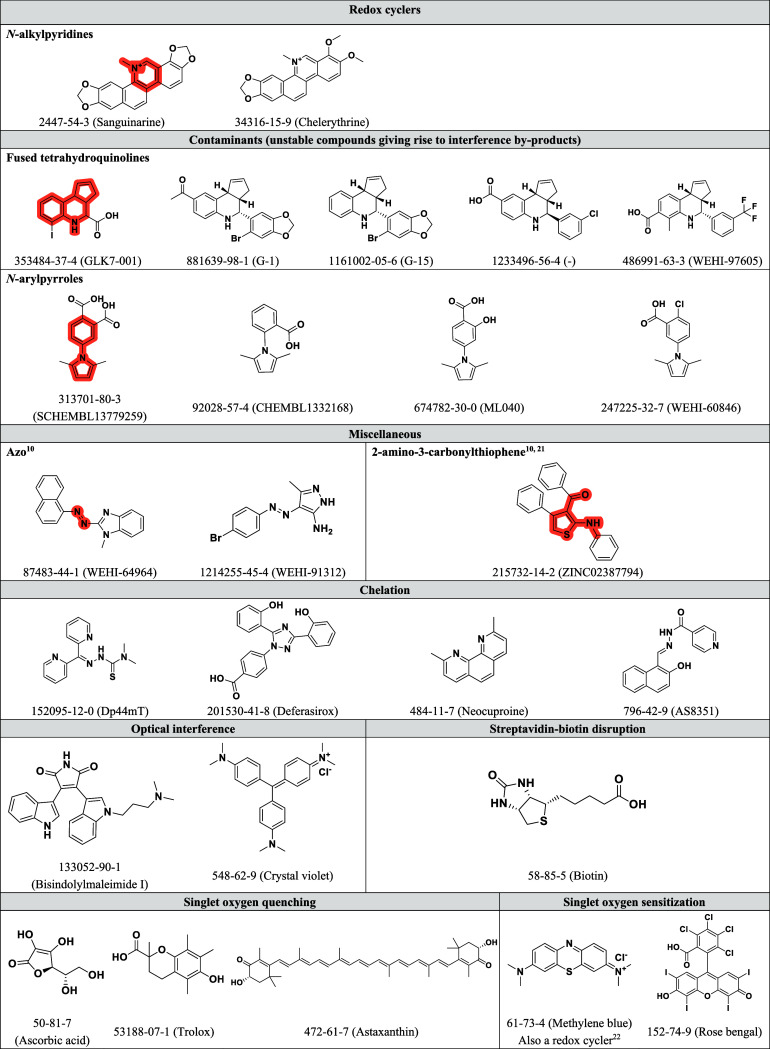
Representative PAINS^a^

a The key structural alert for each PAINS scaffold is highlighted (red) in the exemplary compound. Of note, some compounds can fall into multiple classes due to their varied mechanisms of interference with assays.

#### Electrophiles and Masked Electrophiles

These PAINS are reactive but stable enough to remain intact in screening samples and so may give the impression of being drug-like. As shown in Table 1, we have included alkylidene derivatives of rhodanines (4 compounds), pyrazolones (4), barbiturates (2), which can also exhibit photoreactivity and metal chelation nuisance behavior.^10^ In this category are also included dienones (3) and masked enones such as 2-aminoethylketones that reveal enones through a retro-Michael reaction,^12^ as well as phenolic Mannich bases/Betti bases (3) that similarly form nonspecific electrophilic quinone methides.^13^ Finally, we have included some isothiazolones/disulfides (5) that are frequently reported as useful screening hits yet known to be nonspecifically thiol-reactive.^14−16^

#### Redox Cyclers

We have included representative quinones (2) and catechols (4) that can redox cycle, with a catechol for example interchanging with its ortho-quinoid oxidized form.^17^ Quinones are also electrophilic and represent nuisance behavior via this additional mechanism.^17^ Toxoflavin-type systems are notoriously misleading redox cyclers^18^ and we have included key representatives (4). Representative *N*-alkylpyridines (2), such as sanguinarine and chelerythrine, have been incorporated (Table 1). These benzophenanthridine-based natural products have been documented to trigger rapid apoptosis through redox cycling mechanism, which in turn generates significant cellular levels of reactive oxygen species (ROS), particularly H_2_O_2_.^19,20^

#### Contaminants

Fused tetrahydroquinolines (THQs) decompose to nuisance contaminants in the DMSO solutions^23^ and we have included key examples (5) along with *N*-arylpyrroles (4) that exhibit the same phenomenon (Table 1). In the case of the former, it was observed that THQs containing the cyclopentene ring act as a source of reactivity for fused THQs.^23^ Likewise, anionic polymeric decomposition products of certain *N*-arylpyrroles have been shown to interfere in bioassay signaling.^24^

#### Miscellaneous

We have included azo compounds (2) and a 2-amino-3-carbonylthiophene (Table 1). These are chemotypes we classify as nuisance but underlying mechanisms have yet to be fully elucidated.^10,21,25^

#### Chelators

Compounds that chelate metals are highly problematic, as not only can they encourage retention interfering metal contaminants from prior synthesis, but they can disrupt assay signaling, such as nickel-hexahistidine anchors or via inactivation of functional bioactive metals.^26−32^ Hence, four chelators are included in our curated set (Table 1).^1^

#### Additional Mechanisms

**Figure 2:**
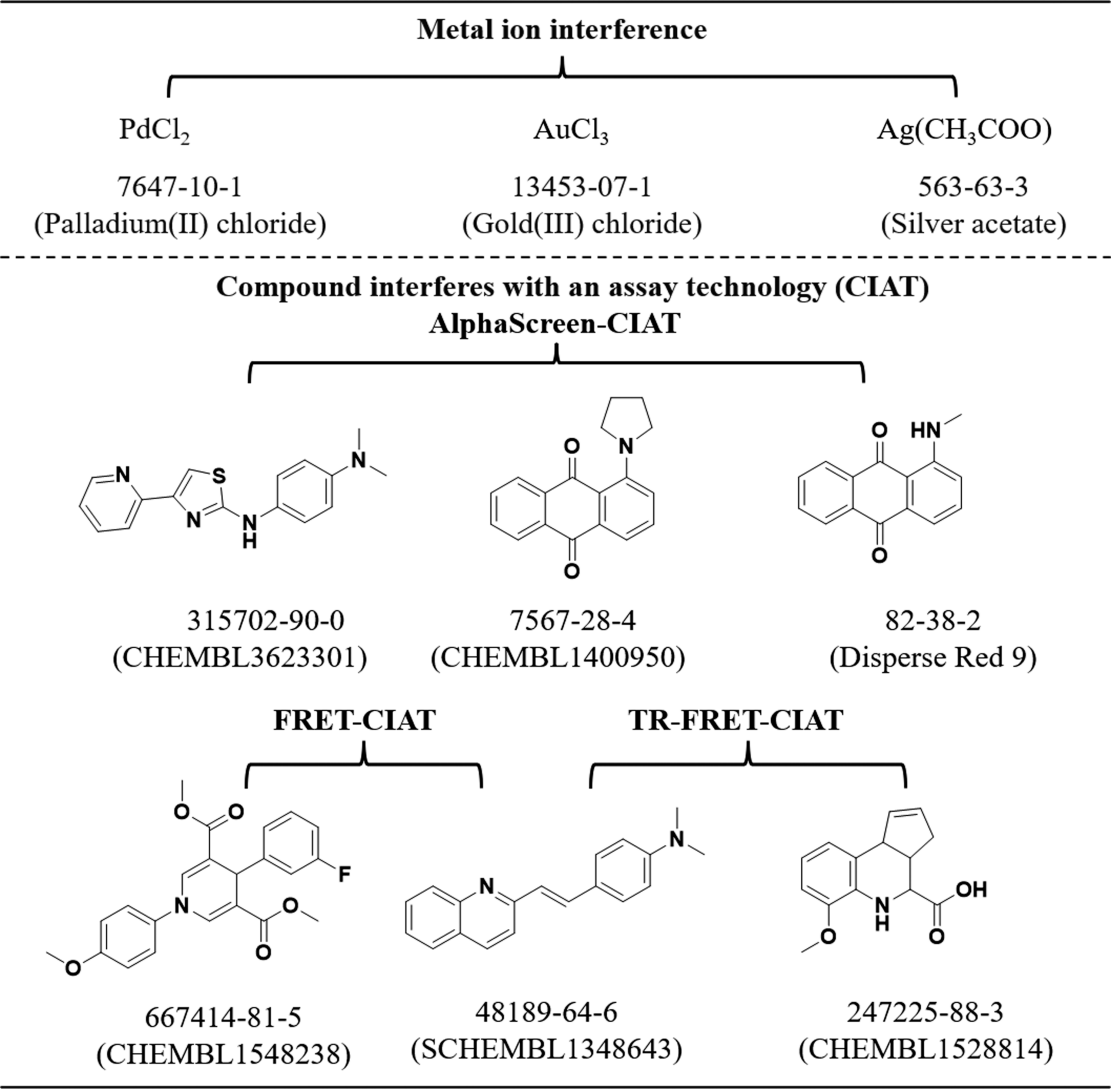
Metal ions and compounds that interfere with assay technologies (CIATs). Shown are three metal salts that mimic on-target activities and compounds that disrupt three widely used HTS assay technologies. The CAS number for each compound is provided along with its name in parentheses. Note the reappearance of quinone and THQ PAINS moieties.

We included compounds (Table 1) causing optical interferences (2), a streptavidin–biotin disruptor (1), compounds involved in singlet oxygen quenching (3), and singlet oxygen sensitizers (2).^1^ Additionally, three representative metal salts that have been reported to interfere with assays, such as the recently reported palladium (Pd) contamination that falsely indicates active hits in a KRAS AlphaScreen assay, are also included.^33^ Moreover, compounds that more broadly interfere in other screening technologies such as FRET (Förster resonance energy transfer) and TR-FRET (time-resolved fluorescence resonance energy transfer) are also selected (Figure 2).^34^

### Phenotypic Nuisance Compounds

Compounds listed in Table 1 may typically also appear as phenotypic actives. There are several compound classes that additionally or uniquely are problematic in cell-based assays.^1^

#### Phenols

**Figure 3:**
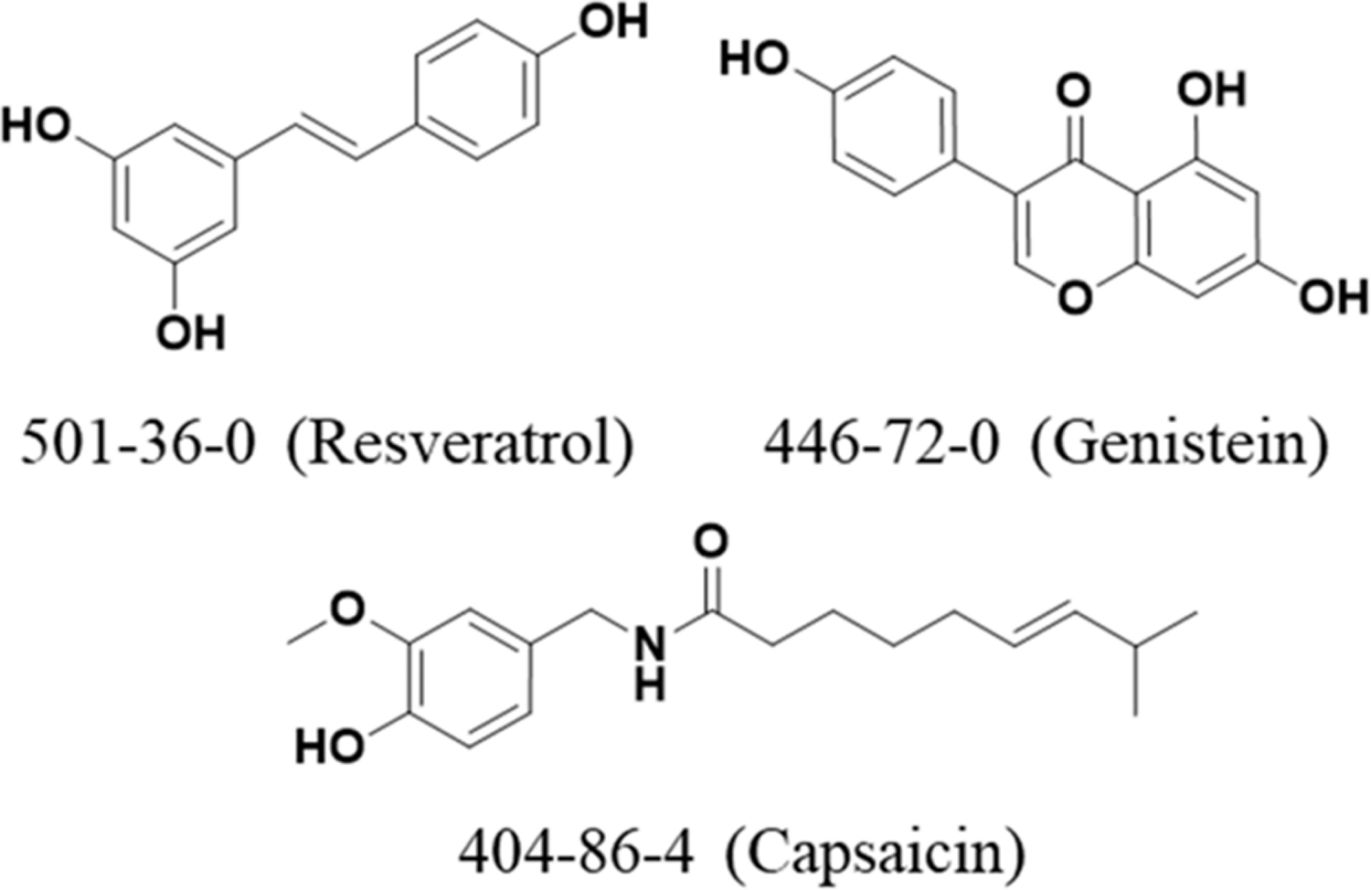
Phenolic nuisance compounds. Displayed are characteristic phenolic phytochemicals that interfere with cell membranes. The CAS number for each compound is provided along with its name in parentheses.

Beyond their ability to undergo redox-cycling and form reactive quinones,^17^ phenols can interfere with cell membranes through a process known as membrane bilayer-mediated mechanism.^35^ This interference occurs even in resorcinols and monophenols, which lack the redox cyclic reactivity found in ortho and para quinoids. Plant secondary metabolites are often rich in phenol groups and so these are prevalent in this category. Such compounds have been termed invalid metabolic panaceas (IMPS)^36^ because of their tendency to pollute the literature with reports of bioactivity that do not progress to anything meaningful. A key report by Ingolfsson^35^ documents some of these subversive behaviors for capsaicin, curcumin (which includes an enone), epigallocatechin gallate (EGCG, also featuring a catechol), genistein, and resveratrol (Figure 3 and Table 1). These phenolic phytochemicals were found to disrupt membrane protein function at low micromolar concentrations by integrating into the interface between the phospholipid bilayer and solution, leading to cell membrane disruption, rather than direct inhibition of specific proteins.^35,37^ Therefore, these compounds are incorporated into our carefully selected nuisance set.

#### Cationic Amphiphilic Drugs (CADs)

**Figure 4:**
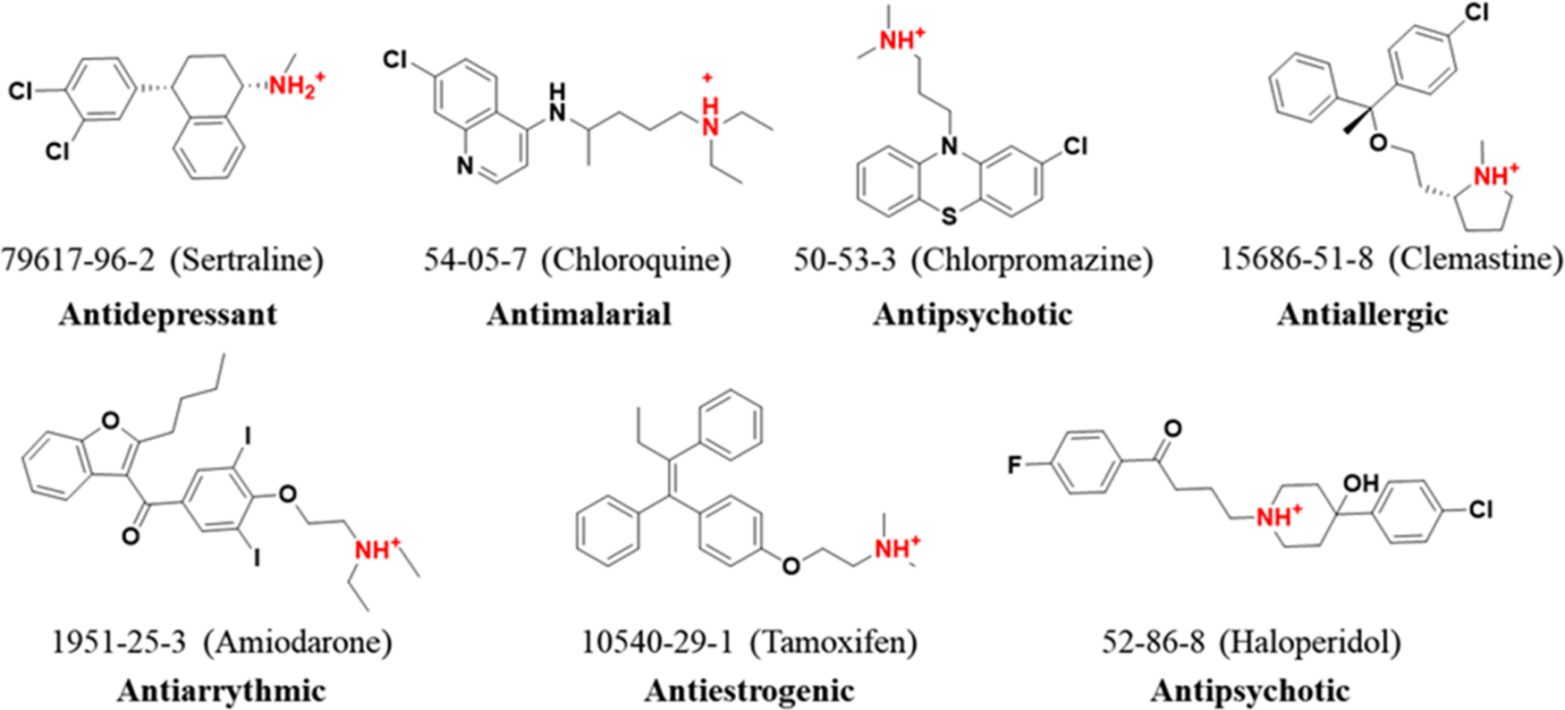
Cationic amphiphilic drugs. Displayed are FDA-approved CADs with various clinical uses known to induce phospholipidosis with their hydrophilic amine headgroup that can be protonated in acidic conditions highlighted in red. The CAS number for each compound is provided along with its name in parentheses.

Many FDA-approved drugs are lipophilic amines that can perturb cellular function at screening concentrations,^1,38^ which are typically far in excess of those that are clinically relevant. Screening libraries with lipophilic amine synthetics are likely to be just as problematic and are frequently reported as phenotypic screening hits. The problems are so extensive that intensive interest in developing predictive algorithms has been generated. This includes adopting a basic physicochemical property model, utilizing cLogP and p*K*_a_ as indicators for compounds that induce phospholipidosis,^39^ as well as our recent progress in large-scale phospholipidosis prediction of tool compounds through the implementation of an explainable machine learning architecture.^40^ Some key CADs such as clemastine, sertraline, amiodarone, chloroquine, chlorpromazine, tamoxifen, and haloperidol, known for inducing phospholipidosis across diverse therapeutic applications^1,38,40^ are included (Figure 4).

#### Pathway-Associated/Cellular Poisons

**Figure 5:**
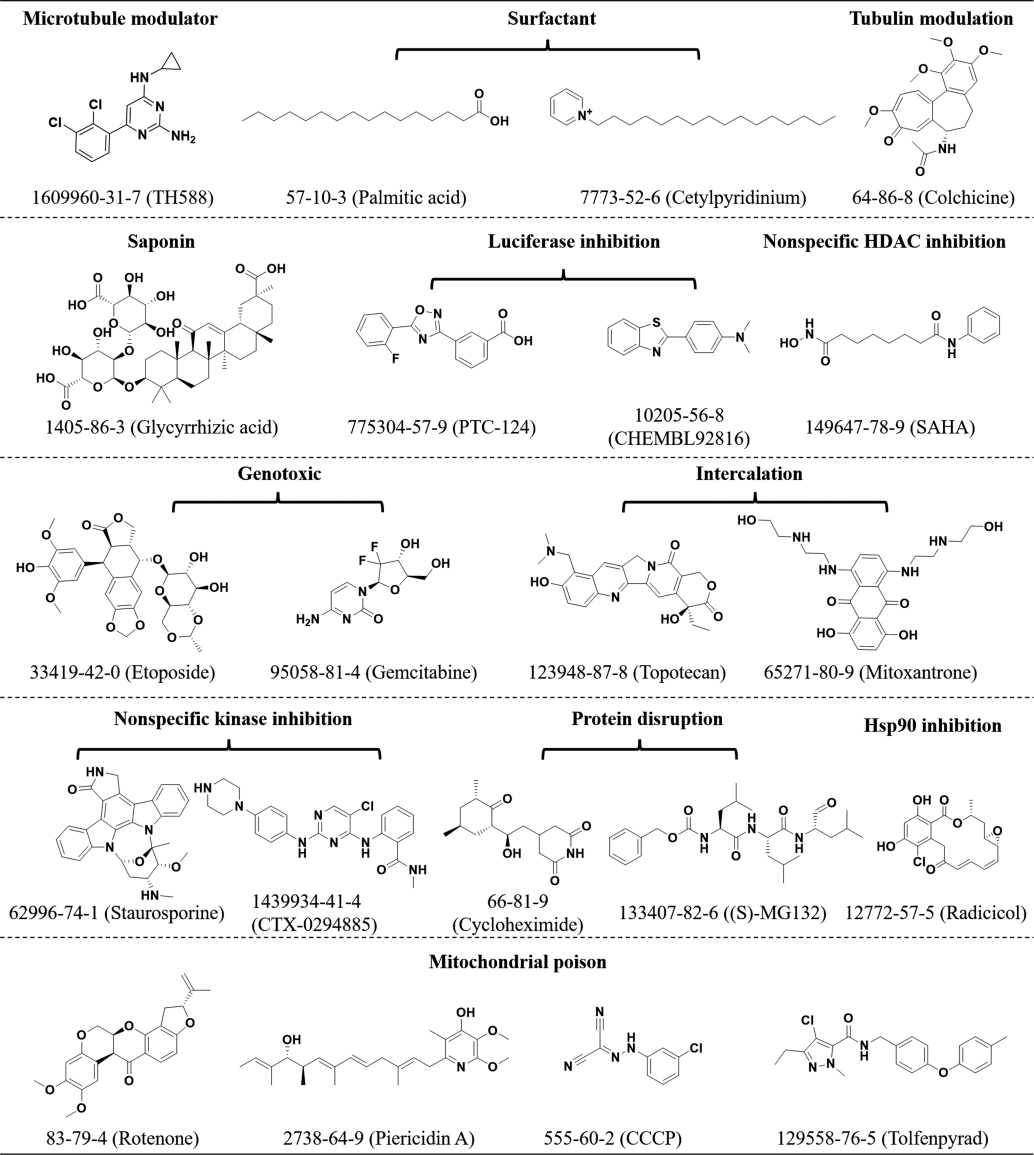
Representative pathway-associated/cellular poisons. Depicted are representative pathway-associated/cellular nuisance compounds. The CAS number for each compound is provided along with its name in parentheses.

In oncology drug discovery in particular, there are pathways that may be influenced by kinase inhibitors, cytoskeletal poisons and genotoxins, and which give an off-target readout that is misinterpreted as an on-target anticancer activity for a protein of interest.^41^ For example, TH588 (Figure 5), functioning as a modulator of microtubules, eradicates cancer cells by activating the mitotic surveillance pathway instead of targeting mut-T homologue 1 (MTH1) and degrading oxidized nucleotides, as originally suggested.^42^ These issues are relevant to other fields such as immunology and parasitology. For instance, tolfenpyrad, which displays potent antiparasitic activity without mammalian cytotoxicity, under respiring conditions reveals itself to be an antilife toxin in being a strong mitochondrial poison.^43,44^ Hence, key representatives of cellular nuisance compounds with varying mechanisms are included (Figure 5). This encompasses a microtubule binder (1), surfactants (2), a tubulin modulator (1), a saponin (1), luciferase inhibitors (2), a pan-HDAC inhibitor (1), genotoxins (2), DNA intercalators (2), pan-kinase inhibitors (2), protein disruptors (2), an Hsp90 inhibitor (1), and mitochondrial poisons (4).^1^

### Reactivity and Relevance to Drug Discovery

It is not that reactive or pharmacologically promiscuous compounds cannot become drugs. Among the cohort of clinically used drugs, there are compounds that bear PAINS moieties and other generally reactive chemistries that may drive or contribute to efficacy. However, these compounds were discovered as potent bioactives in target-agnostic phenotypic assays and their reactive mechanisms subsequently elucidated over a period of years or even decades.^5^ This remains a relevant route to drug discovery, but it does not make sense to utilize such compounds–or the compounds such as those we describe herein–for any precision pharmacology approach. Some covalently acting FDA-approved drugs have been developed in a precision pharmacology and rational sense, and are hallmarked by anticancer ATP-competitive kinase inhibitors. However, here the reactive warhead such as an acrylamide has been introduced subsequent to HTS to identify benign scaffolds followed by optimization and finally warhead incorporation. More recently, covalent-first is an emerging method where small libraries of diverse benign scaffolds, each bearing an invariant warhead such as an acrylamide, are screened to find compounds that label the target of interest. Most typically, only proteins that are structurally enabled are targeted, hits are prioritized based on their ability to selectively and extensively modify a particular cysteine of interest, and specialized assays that can dissect the rate of covalent labeling from noncovalent inhibition are developed, the intention being that labeling driven only through the warhead with little affinity imparted by the rest of the molecule is considered nonprogressable. These concepts are all distinct from those for which the use of our set of nuisance compounds is intended.

## Conclusions

In order to increase the chances of identifying a few on-target screening hits, it is typically recommended for a high-quality screening deck of diverse compounds to number around at least 250,000 compounds.^45^ There is essentially no chance of getting an on-target hit from screening 50–100 such unbiased compounds. For this reason, we believe bioassay hits arising from assembly of a small set of nuisance compounds as listed in Table 1 and Figures 1–5 would be very informative in screening campaigns to help inform target-owners of the types of interference compounds that a target-owner’s assay might be susceptible to. Through identification of promiscuous chemotypes that are likely to interfere in the assay of the target provider, efficient screening hit triage will be facilitated by encouraging focus on less promiscuous compounds which are more likely to represent optimizable bioactives. We include key references in support of specific compound promiscuity (Data set S1) for our **c**ollection **o**f **n**uisance compound**s** (CONs).

Finally, we have ensured that each of these CONS is available from vendors, and further that the entire set is available in assay-ready format (Data set S1)

## Methods

The nuisance compounds in our collection are primarily gathered from literature searches and our previous screening experience.^10^ Reactive compounds are identified using substructure searches with predefined PAINS moieties via the SciFinder service.^10^ For synthetic compounds with reactive PAINS moieties, we examine additional literature evidence to determine how they interfere with assay readouts. We select representative compounds for each PAINS type and include them in our nuisance set.

This set also contains seven FDA-approved cationic amphiphilic drugs, such as sertraline and tamoxifen, which are known to interfere with cellular function by inducing phospholipidosis at screening concentrations.^38^ To enhance the utility of this nuisance compound set, we also include representative cases of metal ions, compounds that interfere with assay technologies, genotoxins, DNA intercalators, mitochondrial poisons, tubulin modulators, saponins, aggregators, surfactants, and protein disruptors. Additionally, Other pathway-associated poisons such as nonspecific HDAC and kinase inhibitors are included. These compounds are primarily curated from literature reported by Dahlin et al.^1^

For each compound in our set, references reporting its activity against biomolecules from the SciFinder and PubChem screening databases are provided.^46^ In total, 103 compounds are included in this set, all of which are available from the Enamine vendor.

## Supporting Information Available

Data set S1: General information of nuisance compound set (targets and references reporting their bioactivities) (XLSX)

## Abbreviations

HTS: high-throughput screening
PAINS: pan-assay interference compounds
TIPT: tetraiodophenolphthalein
ROS: reactive oxygen species
THQs: tetrahydroquinolines
FRET: Förster resonance energy transfer
TR-FRET: time-resolved fluorescence resonance energy transfer
IMPS: invalid metabolic panaceas
EGCG: epigallocatechin gallate
MTH1: mut-T homologue 1
CONs: collection of nuisance compounds
